# Repurposing of anticancer drugs: *in vitro* and *in vivo* activities against *Schistosoma mansoni*

**DOI:** 10.1186/s13071-015-1023-y

**Published:** 2015-08-13

**Authors:** Noemi Cowan, Jennifer Keiser

**Affiliations:** Department of Medical Parasitology and Infection Biology, Swiss Tropical and Public Health Institute, P.O. Box, CH-4002, Basel, Switzerland; University of Basel, P.O. Box, CH-4003, Basel, Switzerland

**Keywords:** Schistosomiasis, Drug repurposing, Drug screening, Protein kinase inhibitor

## Abstract

**Background:**

Drug discovery for the neglected tropical disease schistosomiasis has a high priority. Anticancer drugs, especially protein kinase inhibitors, might serve as a starting point for drug discovery owing to the importance of protein kinases in helminth growth and development. Furthermore, the *Schistosoma mansoni* genome encodes several genes for targets of drugs marketed for human use, including several anticancer drugs.

**Methods:**

In this study, we screened the approved oncology drug set of the National Cancer Institute’s Developmental Therapeutic Program for antischistosomal activity. Drugs were tested *in vitro* against the larval and adult stage of *S. mansoni*. IC_50_ values and albumin binding were determined for active compounds. Lead compounds were tested in the chronic *S. mansoni* mouse model.

**Results:**

Eleven of the 114 compounds tested revealed IC_50_ values ≤ 10 μM against both *S. mansoni* stages. Five of these lost activity against adult *S. mansoni* in the presence of serum albumin. Of 6 compounds studied *in vivo*, the highest activity was observed from two kinase inhibitors trametinib, and vandetanib, which reduced worm burden by 63.6 and 48.1 % respectively, after a single oral dose of 400 mg/kg body weight.

**Conclusion:**

Our study has confirmed that oncology drugs possess antischistosomal activity. There is space for further investigation, including elucidation of the mechanisms of action of schistosome-active cancer drugs, application of different treatment courses, and structure-activity relationship studies for improving drug potency.

## Background

Schistosomiasis is a neglected tropical disease caused by the blood-dwelling fluke of the genus *Schistosoma*. The clinically relevant species are *S. mansoni*, *S. haematobium*, and *S. japonicum*. Approximately 779 million people live at risk of infection, and 230 million are infected [1], causing an estimated 3.3 million disability-adjusted life years (DALYs) [2]. Praziquantel is the sole treatment against all three species. The lack of drugs in the discovery pipeline highly encourages efforts to identify an alternative treatment of schistosomiasis, in anticipation of praziquantel resistance [3, 4]. Drug repurposing is an efficient tool to find new drugs against helminthiases, reducing time and costs of drug research and development [5].

In recent years, imatinib (Gleevec®), a kinase inhibitor used to treat chronic myeloid leukemia, gained attention in the field of antischistosomal drug research due to its dose- and time-dependent effect on *S. mansoni in vitro* [6]. It has been observed that imatinib causes degenerative changes in the gonads and gastrodermis of schistosomes [7]. Furthermore, protein kinase inhibitors interfere with essential developmental steps in the biology of schistosomes [6, 8].

We have recently identified *N,N*’-diarylureas as a new chemical class potent against *S. mansoni* [9]. A subsequent structure-activity relationship (SAR) study revealed *N,N’*-diarylureas and *N*-phenyl benzamides as the relevant pharmacophores for antischistosomal drug activity [10]. These pharmacophores are also present in some marketed anticancer drugs, such as sorafenib and ponatinib.

The Developmental Therapeutics Program (DTP) of the National Cancer Institute (NCI) (USA) offers drug repositories free of charge to endorse preclinical research (https://dtp.nci.nih.gov/repositories.html); among which is a set of the US Food and Drug Administration (FDA)-approved anticancer drugs.

There are 54 drugs for human use on the market which exert their pharmacological effect on 26 drug targets which are also encoded by the genome of *S. mansoni* [11]. Fourteen of those drugs are part of DTP’s oncology drug set.

The aim of this study was to evaluate DTP’s oncology drug set of 114 FDA-approved drugs for antischistosomal activity. We tested the drugs first on the larval stage of *S. mansoni* (schistosomula), followed by screening of larvae-active compounds against adult worms. IC_50_ values were then determined against adult *S. mansoni,* and the influence of protein binding on drug activity was assessed using physiological amounts of serum albumin. The most active compounds were subsequently tested in *S. mansoni*-infected mice.

## Methods

### Drugs and media

The cancer drug library used for schistosome *in vitro* assays was gratefully received in June 2014 from the DTP/NCI as 10 mM stock solutions (20 μl) in dimethyl sulfoxide (DMSO) in 96-well plates. Hit compounds were ordered as solid compounds from DTP, and dissolved in DMSO to 10 mM stock solutions. Bosutinib was not available from DTP and was therefore purchased from Sigma-Aldrich. For *in vivo* studies, solid afatinib, bosutinib, ponatinib, trametinib, and vandetanib were purchased from AkScientific. Sunitinib was purchased from VWR as a 100 mM solution in DMSO.

Medium 199, and RPMI 1640 were purchased from Life Technologies. Heat-inactivated fetal calf serum (FCS), penicillin, and streptomycin were purchased from LuBioScience.

### Mouse infection and maintenance

Rodent experiments were authorized by the Canton Basel-Stadt, Switzerland (license no. 2070).

Female NMRI mice, 3-weeks of age, were purchased from Charles Rivers, Germany. After a 1-week adaptation period, mice were infected with cercariae collected from *S. mansoni*-infected intermediate host snails *(Biomphalaria glabrata)*, by subcutaneous injection with 100 cercariae [12]. Mice received rodent food and water ad libitum and were maintained with a 12-h light/dark cycle, at 22 °C and 50 % humidity.

### Larval schistosome drug assay

*S. mansoni* cercariae were collected from *S. mansoni*-infected *B.glabrata*, and mechanically transformed to newly transformed schistosomula (NTS) [13]. After a resting period of 12–24 h (37 °C, 5 % CO_2_), drugs were tested for NTS activity at a concentration of 33.3 μM in Medium 199 supplemented with 5 % FCS, 200 U/ml penicillin, and 200 μg/ml streptomycin, and prepared in 96-well flat-bottom plates with 100 NTS per well. NTS incubated with the equivalent volume of drug-free DMSO (0.3 %) served as control. NTS were evaluated 24, 48, and 72 h after incubation via microscopic read out (80–120× magnification; Zeiss; Germany), using a scoring scale from 3 (normal viability, morphology, and granularity) to 0 (no motility, changed morphology, and granularity). Drugs with an activity of ≥ 50 % after 24 h, and/or 90 % after 72 h, and a drug effect on adult schistosomes of ≥ 80 % after 24 h and/or 90 % after 72 h at 33.3 μM, were tested at six different concentrations ranging from 0.14 to 33.3 μM using a 3-fold dilution series for IC_50_ determination. All assays were performed in duplicate and repeated once [10].

### Adult schistosome drug assay

Adult schistosomes were collected from mice with a chronic *S. mansoni* infection (7-week-old) by dissection of the mesenteric veins. Drugs were tested at 33.3 μM in RPMI 1640 culture medium supplemented with 5 % FCS, 100 U/ml penicillin, and 100 μg/ml streptomycin, and prepared in 24-well flat-bottom plates. Three flukes of both sexes were put into the wells, incubated at 37 °C, and 5 % CO_2_, and scored (in the same manner as described for NTS) after 1, 24, 48, and 72 h. Drugs revealing activity against NTS, and adult schistosomes (as explained above), were assessed for their IC_50_, using 3-fold serial dilutions resulting in five different concentrations ranging from 0.41 to 33.3 μM, and scored 4, 24, 48, and 72 h post incubation. IC_50_ determinations were performed in duplicate, and repeated once [12]. For compounds exhibiting an IC_50_ < 33.3 μM, IC_50_s were determined using culture medium supplemented with 45 g/l bovine serum albumin (AlbuMax® II Lipid-Rich BSA, Gibco): the physiological albumin concentration in humans [14].

### Preclinical and clinical data from FDA and EMA

FDA and European Medicines Agency (EMA) data sheets were used to retrieve drug information such as the maximal plasma concentration (C_max_), plasma half-life (t_1/2_), nonclinical toxicology (lethal single oral dose LD_50_), indication, mechanism of action, and dosage.

### *In vivo* adult schistosome drug assay

For oral application, the drugs were dissolved in 7 % Tween 80 and 3 % ethanol in water (v/v/v), with the exception of sunitinib, which was used as obtained. Groups of 4 mice harboring a chronic *S. mansoni* infection were treated with a single oral dose of 400 mg/kg body weight, or 200 mg/kg for afatinib due to its low LD_50_ (382–763 mg/kg in mice) [15]. A control group of 8 mice was left untreated. Three weeks post treatment, the mice were euthanized, and schistosomes residing in the mesenteric veins and the liver were counted and sexed.

### Statistics

Drug effects on schistosomes were determined with the scores of parasites exposed to drug, and the score of the controls. For IC_50_ and r value (linear correlation coefficient) determination, the dose-response was calculated with CompuSyn (version 3.0.1; ComboSyn), as described previously [10]. *In vivo* worm burden reductions (WBR) were calculated with the number of worms found in treated mouse groups compared to the control group [10]. *P*-values were calculated using the Kruskal-Wallis test (Stats direct statistical software version 2.8.0).

## Results

### *In vitro* studies

#### *In vitro* activities against NTS

**Fig. 1:**
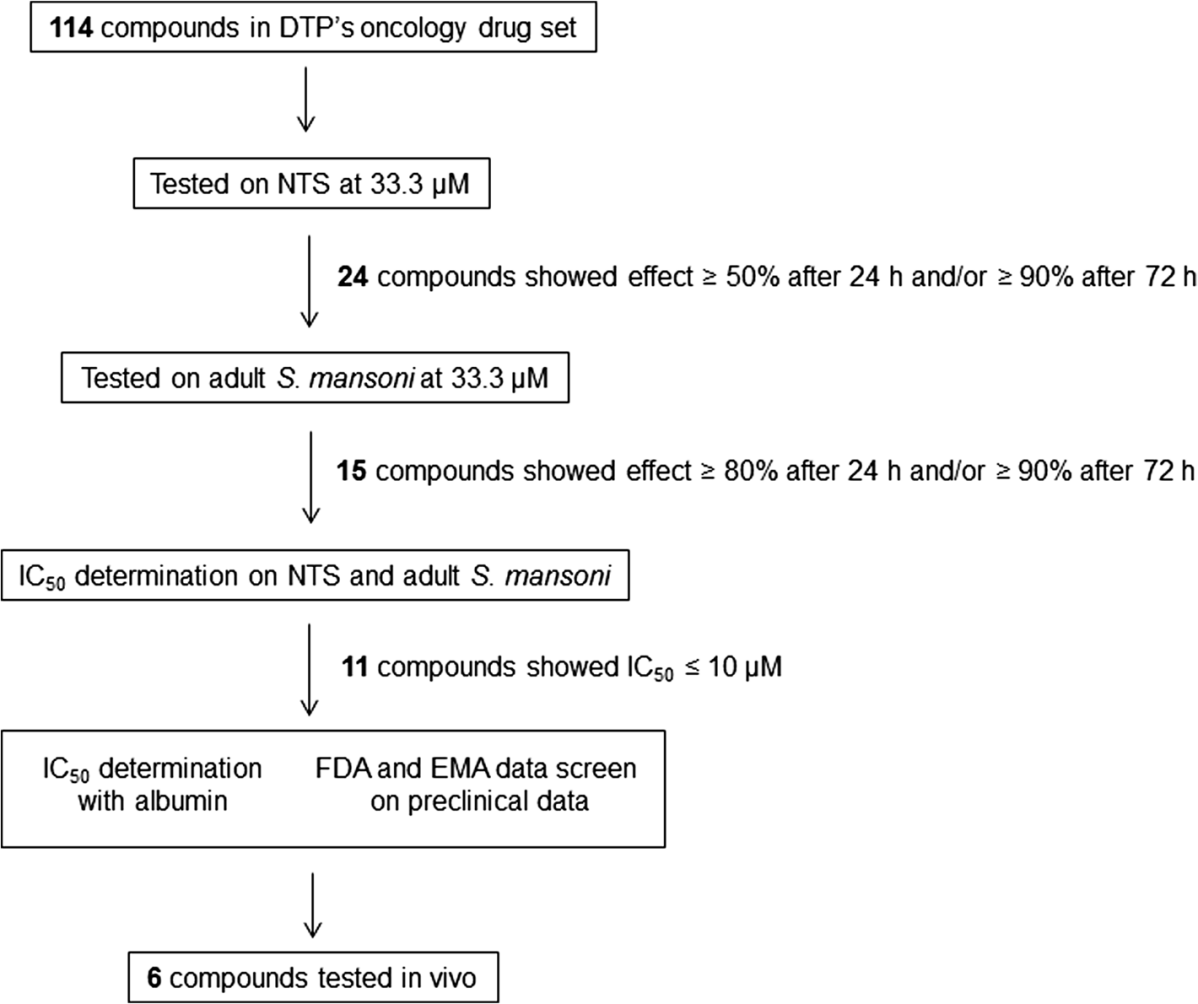
Experimental flow

DTP’s oncology drug set was first tested at 33.3 μM against NTS (Fig. 1). Twenty-four drugs showed an effect ≥ 50 % after 24 h, and/or ≥ 90 % after 72 h. The most active drugs were crizotinib, ponatinib, and tamoxifen citrate, killing NTS in less than 1 h. Afatinib, idarubicin hydrochloride, regorafenib, sorafenib, and temsirolimus were lethal to NTS within 24 h, everolimus and sirolimus within 48 h, and bosutinib, daunorubicin, and vandetanib within 72 h (Table 1).

**Table 1:** IC_50_ values of anticancer drugs against larval (NTS) and adult stages of *S. mansoni*

Compound	NTS: IC_50_ value [μM]	Adult *S. mansoni*: IC_50_ value [μM]	Adult *S. mansoni*: IC_50_ value in the presence of albumin [μM]
Regorafenib	6.9	1.0	>> 33.3
Ponatinib	0.2	1.1	20.2
Sorafenib	4.1	1.1	>> 33.3
Bosutinib	0.8	1.5	14.8
Afatinib	0.8	1.8	9.9
Sunitinib	1.0	1.8	10.6
Crizotinib	0.3	2.3	18.8
Trametinib	4.6	4.1	21.0
Tamoxifen citrate	2.0	5.7	>> 33.3
Gefitinib	7.8	8.3	>> 33.3
Cabozantinib	19.3	9.0	>> 33.3
Celecoxib	41.9	9.4	>> 33.3
Vandetanib	0.9	9.5	>> 33.3
Idarubicin HCl	12.7	16.2	>> 33.3
Mechlorethamine HCl	> > 33.3	84.8	Not done

#### *In vitro* activities against adult *S. mansoni*

The 24 NTS-active compounds were subsequently tested on adult *S. mansoni* at 33.3 μM. Tamoxifen citrate was the most active compound, killing all adult worms within 1 h. Afatinib, ponatinib, and sorafenib caused death of all adults within 24 h, whereas adult worms incubated with bosutinib and idarubicin hydrochloride were dead after 72 h. In total, 15 compounds were classified as active against adult *S. mansoni*, characterized by an effect ≥ 80 % after 24 h, and/or ≥ 90 % after 72 h (Table 1).

### Determination of IC_50_ values

These 15 compounds were investigated further by assessing their IC_50_ values against adult worms and NTS. Eleven compounds (structures depicted in Table 2) revealed high activities (IC_50_ ≤ 10 μM) against both stages after 72 h.

**Table 2:** Chemical structures of lead compounds

		
afatinib	bosutinib	crizotinib
		
gefitinib	ponatinib	regorafenib
		
sorafenib	sunitinib	tamoxifen
		
trametinib	vandetanib	

However, IC_50_ values increased in the presence of albumin. In medium supplemented with 45 g/L BSA, only 6 compounds showed activity (IC_50_ of 9–21 μM against adult *S. mansoni*), namely afatinib, bosutinib, crizotinib, ponatinib, suntinib, and trametinib. For the remaining compounds tested, no IC_50_ could be calculated due to lack of activity.

### Preclinical and clinical data

Data on pharmacokinetic parameters, toxicity, and other information of interest accessible from the FDA and the EMA (summarized in Table 3) were consulted for the 11 compounds characterized by an IC_50_ ≤ 10 μM against both stages in order to select good *in vivo* candidates. With regards to potential drug exposure time, vandetanib has a very long half-life (19 days in humans), which we considered an advantageous feature for killing parasites that reside in the bloodstream.

**Table 3:** FDA and EMA drug description of anticancer drugs of lead compounds

Drug	C_max_ Single oral dose (or otherwise as indicated)	t_1/2_ Single oral dose (or otherwise as indicated)	LD_50_ Single oral dose toxicity	Indication	Mechanism of action	Dosage	Reference (health agency)
Afatinib (GIOTRIF®)	NA	37 h (after repeated dosing given to patients)	NA	Metastatic non-small cell lung cancer	Irreversible inhibitor of tyrosine kinase autophos-phorylation	40 mg/day	FDA
	397 nmol/l (1×8 mg/kg given to rats)	4.5 h (1×8 mg/kg given to rats)	382–763 mg/kg (mice)				EMA
Bosutinib (BOSULIF®)	0.2 μg/ml (500 mg given to patients on 15 consecutive days)	22 h (patients; dose not indicated)	NA	Chronic, accelerated, or blast phase Ph + chronic myelogenous leukemia	Tyrosine kinase inhibitor	500 mg/day	FDA
	NA	2.5–5.4 h (mice and rats; dose not indicated)	>2000 mg/kg (mice and rats)				EMA
Crizotinib (XALKORI®)	100–135 ng/ml (250 mg given to patients)	42 h (250 mg given to patients)	>500 mg/kg (rats)	Metastatic non-small cell lung cancer	Tyrosine kinase inhibitor	2×250 mg/day	FDA
	NA	5.8–13 h (rats; dose not indicated)	NA				EMA
Gefitinib (IRESSA®)	NA	48 h (healthy volunteers; dose not indicated)	NA	Non-small cell lung cancer	Multiple tyrosine kinase inhibitor	250 mg/day	FDA
	1 μg/ml (after 20 mg/kg given to rats); 0.1 μg/ml (after 250 mg/kg given to healthy volunteers)	10 h (rats; dose not indicated); 30 h (after 250 mg/kg given to healthy volunteers)	Around 2000 mg/kg (rats); >1000 mg/kg (dogs)				EMA
Ponatinib (INCLUSIG®)	6 h (patients; dose not indicated)	24 h (patients; dose not indicated)	>2000 mg/kg (mice)	Chronic myeloid leukemia	Tyrosine kinase inhibitor	45 mg/day	FDA
	4 h (patients; dose not indicated)	22 h (patients; dose not indicated)	NA				EMA
Regorafenib (STIVAGRA®)	12.5 μg/ml (after 160 mg given to patients)	24 h (after 160 mg given to patients)	NA	Metastatic colon cancer	Multiple protein kinase inhibitor	160 mg/day for first 21 days of a 28-day cycle	FDA
	3.96 mg/l (multiple treatment: 160 mg/day for 3 weeks given to patients)	2 h (multiple treatment; 160 mg/day for 3 weeks given to patients)	>250 mg/kg (mice and rats)				EMA
Sorafenib (NEXAVAR®)	NA	25–48 h	NA	Liver, kidney, thyroid cancer	Multiple protein kinase inhibitor	2×400 mg/day	FDA
	0.55 mg/l (after 400 mg given to patients)	22.3 h (after 400 mg given to patients)	>1460 mg/kg (mice and rats)				EMA
Sunitinib (SUTENT®)	NA	40–60 h (parent drug in healthy volunteers; 80–110 h (active metabolite in healthy volunteers (dose not indicated)	NA	Gastrointestinal stromal tumor, renal cell carcinoma, well-differentiated pancreatic neuroendocrine tumors	Inhibitor of multiple receptor tyrosine kinases	50 mg/d for the first 28 days of a 42-day cycle	FDA
	NA	NA	>500 mg/kg (mice and rats)				EMA
Trametinib (MEKINIST®)	NA	Estimated: 3.9–4.8 days (patients; dose not indicated)	NA	Unresectable or metastatic melanoma with BRAF V600E or V600K mutations	Kinase inhibitor	2 mg/day	FDA
	22.2 ng/ml (steady state after 2 mg/daygiven to healthy volunteers)	5.3 days (healthy volunteers; dose not indicated)	NA				EMA
Tamoxifen citrate (NOLVADEX®)	40 ng/ml (after 20 mg given to rats)	5–6 days (after 20 mg given to rats)	NA	Breast cancer	Nonsteroidal antiestrogen	NA	FDA
	NA	NA	NA			20 mg/day	EMA
Vandetanib (CAPRELSA®)	NA	NA	NA	Medullary thyroid cancer	Multiple tyrosine kinase inhibitor	300 mg/day	FDA
	NA	19 days (after 300 mg given to healthy volunteers)	NA				EMA

*NA* Not available on the data sheets of the according health agency

### Activity in *S. mansoni*-infected mice

Afatinib, bosutinib, ponatinib, sunitinib, trametinib, and vandetanib were chosen for *in vivo* studies based on their *in vitro* activity against schistosomes, and review of the literature. Of note, since crizotinib was not affordable, it was not tested *in vivo*. Drugs were orally applied to mice in a single dose of 400 mg/kg body weight; except for afatinib, which was administered at a single dose of 200 mg/kg, given its lower LD_50_. Trametinib, and vandetanib had the highest WBRs of 63.6 %, and 48.1 % respectively (*p*-value > 0.05). The remaining compounds were only marginally, or not at all efficacious, with WBRs between 0–27.5 % (Table 4).

**Table 4:** *In vivo* worm burden reductions after a single oral dose of 200 mg/kg (afatinib) or 400 mg/kg body weight (remaining drugs) to mice harboring a chronic *S.mansoni* infection

Drug	Number of mice treated	Average worm count (SD)	WBR [%]
Control^1^	8	20.4 (12.4)	-
Control^2^	8	23.0 (18.4)	-
Trametinib^1,2^	5	8.1 (4.1)	63.6
Vandetanib^1,2^	5	11.3 (8.4)	48.1
Afatinib^1^	4	14.8 (11.1)	27.5
Ponatinib^1^	3	16.6 (12.9)	18.6
Sunitinib^1^	4	22.5 (8.5)	2.2
Bosutinib^1^	4	25.3 (4.0)	0

*P* value of all WBRs was > 0.05; Values in superscript refer to the corresponding control group

*SD* standard deviation

## Discussion

New drugs are needed to treat the neglected tropical disease schistosomiasis. In the present work, we applied a repurposing strategy using a set of FDA-approved anticancer drugs. This library was chosen given proposed overlaps in mechanism of action, active pharmacophores, and matches of human drug targets found in the genome of *S. mansoni* [6, 11, 16].

Because repurposing builds upon previous research and development efforts, new antischistosomal drugs could quickly advance into clinical testing, greatly diminishing the huge costs of drug development [5]. However, it is worth reflecting on the selected library. Anticancer agents are often characterized by the occurrence of numerous and severe adverse events. Since anthelmintic chemotherapy consists typically of a single dose [17], the adverse events occurring during the intensive multiple-dose regimens of cancer chemotherapy [18], would probably not occur. This encourages studying the anthelmintic properties of anticancer drugs further and in more detail. However, the health-risk benefits of repurposing cancer drugs should be evaluated on a case-by-case basis.

We identified 11 cancer drugs in this work with high *in vitro* activity against adult and larval *S. mansoni*. It is worth highlighting that 10 of these drugs are protein kinase inhibitors, which have been suggested as potentially interesting antischistosomal drug discovery candidates, since protein kinase inhibitors can interfere with signaling pathways in schistosome development [6]. The exact mechanism(s) of action of these drugs on schistosomes remain yet to be elucidated, although apoptosis might be involved, due to the fact that many protein kinase inhibitors induce apoptosis [19].

Six of these compounds maintained their antischistosomal activity when exposed to serum albumin - the predominant plasma protein in humans [14] - while the antischistosomal activity of 5 lead candidates was strongly negatively influenced by serum albumin. The loss of *in vitro* antischistosomal activity of imatinib (a protein kinase inhibitor, which did not progress further in our screens) in the presence of alpha-1-acid glycoprotein, serum albumin, and especially with a combination of both, has been described recently [20]. The influence of alpha-1-acid glycoprotein on the *in vitro* activity of our lead compounds was not studied in the present work since binding to this protein might play a more crucial role in rodents than in humans, as described below.

*In vivo* drug efficacy determination, based on our *in vitro* findings (taking into account the loss of activity in the presence of albumin), as well as a literature review on preclinical and clinical data of these drugs (Table 3), revealed two kinase inhibitors: trametinib, and vandetanib, with moderate WBRs of 63.6 and 48.1 % respectively. Vandetanib’s efficacy was somewhat surprising, since the addition of albumin to the *in vitro* IC_50_ determination led to inactivity of the drug. Protein binding to serum albumin, and apha-1-acid glycoprotein (90 %) was also highlighted by the manufacturer [21]. However, trametinib and vandetanib have a high bioavailability of 100 % [22], or > 90 % [23] in rodents. Additionally, both drugs have long half-lives: 3 days in rats and mice for trametinib [24]; and 28 h in mice for vandetanib [25]. In humans, both trametinib and vandetanib also have exceptionally long half-lives: 4.2 days (3.9 – 4.5 days) [26], and 19 days [27] respectively. The high bioavailabilities combined with the long half-lives might therefore outweigh the negative influence of protein binding on the antischistosomal activity, and explain the efficacy against *S. mansoni* in the mouse model. There might even be a possibility for higher efficacy of vandetanib in humans, since the alpha-1-acid glycoprotein homeostasis is species-dependent. While this serum protein in humans increases 2–5-fold upon inflammatory processes, the increase in mice is 30–40-fold, which is a crucial difference when alpha1-acid glycoprotein-sensitive drugs are being evaluated [16].

Interestingly, none of the 14 drugs, for which the genes of the corresponding human drug targets also exists in *S. mansoni* [11], revealed noteworthy antischistosomal activity (IC_50_ > 33.3 μM) (data not shown). Only temsirolimus, and sirolimus killed NTS within 24 or 48 h respectively; but neither of the two reduced the viability of adult *S. mansoni* considerably. However, we would like to highlight that our drug activity assessments are based on alterations on the parasite phenotype. We did not determine the effect on schistosome development, such as the reproductive organs, or egg production and expulsion, which might be affected by the 14 drugs.

When comparing our *in vitro* results with those of other research groups, differences in drug activity are notable. Under our screening conditions, at 33.3 μM, and 72 h drug exposure, imatinib showed <70 % activity against schistosomula, and 76 % against adult schistosomes, while all worms were still moving. In contrast, Beckmann and Grevelding (2010) described the activity of imatinib (72 h postincubation) to be fatal for 30 % of all adult worms after incubation at 10 μM, or 63 % after incubation at 50 μM [6]. According to Katz *et al* (2013), 6 % of worms died after incubation with imatinib (25 μM for 24 h), followed by 48 h in drug-free culture medium [28]. The reason for the different survival rate is not clear, but might originate from differences in drug susceptibilities of different *S. mansoni* strains (Puerto Rican; Luiz Evangelista versus Liberian).

## Conclusion

In summary, the oncology drug set revealed several *in vitro*-active drugs against *S. mansoni*; of which two (trametinib, and vandetanib) were also moderately active *in vivo*. There is room to further investigate trametinib’s and vandetanib’s potential as antischistosomal drugs, including elucidation of mechanisms of action, application of different treatment courses, and structure-activity relationship studies.
